# Urinary beta-trace protein gene expression analysis in type 2 *diabetes mellitus* patients

**DOI:** 10.1590/S1679-45082017AO4012

**Published:** 2017

**Authors:** Marcelo Rodrigues Bacci, Beatriz da Costa Aguiar Alves, Marina Romera Cavallari, Ligia Ajaime Azzalis, Ross Martin de Rozier-Alves, Matheus Moreira Perez, Ethel Zimberg Chehter, Edimar Cristiano Pereira, Fernando Luiz Affonso Fonseca

**Affiliations:** 1 Faculdade de Medicina do ABC, Santo André, SP, Brazil.; 2 Universidade Federal de São Paulo, Diadema, SP, Brazil.

**Keywords:** Beta-trace protein, Prostaglandins D/biosynthesis, *Diabetes mellitus*, type 2, Gene expression, Biomarkers/urine, Proteína beta-traço, Prostaglandinas D/biossíntese, *Diabetes mellitus* tipo 2, Expressão gênica, Biomarcadores/urina

## Abstract

**Objective:**

To evaluate the gene expression of beta-trace protein in urine of diabetic patients, with no reduction in glomerular filtration rate, which was defined as below 60mL/min/1.73m^2^.

**Methods:**

Type 2 *diabetes mellitus* patients were recruited, and a group of non-diabetic individuals served as control. Beta-trace protein gene expression was analyzed by quantitative PCR. Blood samples were collected to establish glucose levels and baseline kidney function. Accuracy was analyzed using ROC curves.

**Results:**

Ninety type 2 *diabetes mellitus* patients and 20 non-diabetic individuals were recruited. The area under the curve was 0.601, sensitivity of 20% and specificity of 89.47%. Among diabetic participants, 18% showed an expression above the cutoff point.

**Conclusion:**

These results of accuracy of beta-trace protein gene expression in urine of diabetic patients are promising, although they did not achieve a higher area under the curve level.

## RESUMO

**Objetivo:**

Avaliar a expressão do gene da proteína beta-traço na urina de pacientes diabéticos, sem redução na taxa de filtração glomerular, definida como abaixo de 60mL/min/1,73m^2^.

**Métodos:**

Foram recrutados pacientes com *diabetes mellitus* tipo 2, e um grupo de indivíduos não diabéticos serviu como controle. A expressão do gene da proteína beta-traço foi analisada por PCR quantitativa. Amostras de sangue foram coletadas para estabelecer níveis de glicemia e função renal inicial. A acurácia foi analisada utilizando curvas ROC.

**Resultados:**

Foram recrutados 90 pacientes com *diabetes mellitus* tipo 2 e 20 não diabéticos. A área sob a curva foi de 0,601, com sensibilidade de 20% e especificidade de 89,47%. Entre os diabéticos, 18% apresentaram expressão acima do ponto de corte.

**Conclusão:**

Estes resultados de acurácia da expressão do gene da proteína beta-traço na urina de pacientes diabéticos são promissores, apesar de não terem atingido um nível alto na área sob a curva.

## INTRODUCTION

Prostaglandin D-synthase, also known as lipocalin-type beta-trace protein (BTP), is a low-molecular-weight protein of 168 amino acids, encoded on chromosome 9. Beta-trace protein is widely present in a variety of tissues and body fluids. The molecular weight of BTP varies among its different isoforms, ranging from 23 to 29kDa. These isoforms are produced through a post-translational process of N-glycosylation, with the lower-weight isoforms being found mainly in cerebrospinal fluid, and those of higher molecular weight in the blood and urine.^(^ ^1^ ^,^ ^2^ ^)^

The two main serum measurements used in the diagnosis of diabetes are blood glucose and the glycated hemoglobin. As with other chronic diseases, the search for more sensitive and specific biomarkers of diabetes is important to enable earlier interventions, to minimize the progression of the disease and its complications in potential target organs, such as kidneys, heart and brain.

One of the challenges in discovering new biomarkers lies in dosing, which should be simple and practical to facilitate its clinical use, but can potentially interfere in the determination of quantities of the biomarker. This is especially true in the case of BTP, given that the concentration of BTP is high in sites that are undergoing inflammatory processes or, in the case of the brain, suffering from trauma. As such, studies of BTP as a potential marker of renal dysfunction have focused on cases in which elevated levels of BTP have been found in blood and urine. In cases in which high levels of urinary BTP are verified, it is reasonable to consider the possibility kidney damage.^(^ ^3^ ^)^ As a predictor of early renal dysfunction, urinary BTP levels showed a better predictive value for kidney disease than other traditional biomarkers.^(^ ^4^ ^)^

## OBJECTIVE

To evaluate the expression of beta-trace protein gene in the urine of patients with diabetes, who do not present reduction in glomerular filtration rate, defined as below 60mL/min/1.73m^2^.

## METHODS

Patients diagnosed with type 2 *diabetes mellitus* (T2DM) with a history of at least 5 years of disease determined in accordance with the criteria established by the American Diabetes Association were recruited between 2013 and 2014 among nephrology and endocrinology outpatients in the metropolitan area of São Paulo. The exclusion criteria included the existence of infection, an active malignant neoplasm at the moment of enrolment, presence of acquired immunodeficiency syndrome, and a diagnosis of end-stage renal disease, under conservative treatment or dialysis.

This cross-sectional study was designed to evaluate the expression of BTP during diabetes, and did not involve changes in the treatment regimen of the participants. The two groups studied were classified by the presence or absence of T2DM.

The inclusion criteria for the Control Group were absence of diagnosis of diabetes and chronic kidney disease, and no family history of diabetes and chronic kidney disease.

Samples of blood and urine were collected at the time of recruitment to analyze levels of fasting blood glucose, glycated hemoglobin, serum creatinine and urinary BTP.

Urinary BTP gene expression was measured by isolating urinary RNA (1*μ*g, initially), which was then converted into first-strand cDNA with the aid of SSIII First Strand qPCR Supermix (Invitrogen, catalog number 11,752,050), according to the manufacturer's protocol.

The expression of the specific BTP gene was assessed by quantitative real-time polymerase chain reaction (RT-qPCR). To standardize and determine the relative expression of the target gene, the reference gene glyceraldehyde 3-phosphate dehydrogenase (GAPDH) was also expressed. The specific primers of the target and reference genes were designed with the help of the software Primer3 Input 0.4.0 and were checked for their specificity by the Primer-BLAST program.

For RT-qPCR, an Applied Biosystems 7500 Real Time PCR Systems (Applied Biosystems, Foster City, USA) thermal cycler was used. Each sample had a final volume of 15*μ*L, containing 1 x SYBR Green mix (Quantitec SYBR Green PCR kit, QIAGEN Catalog no. 204 054), 10pmol of each specific primer and 2*μ*L of 10x-diluted cDNA. The parameters of amplification included an initial hot start at 95°C for 15 seconds, and 60°C for the primer sequence.

To verify differences in urinary beta-trace protein gene expression between groups, the mean and standard deviation were analyzed using the Student's *t* test for independent samples. The median and first and third quartiles were analyzed using the Mann-Whitney test.

To verify the expression of urinary BTP gene above and below the cutoff point of 6.34, as established in the literature, the relative frequency was used. The verification of a possible association between the quantities expressed between the two groups was performed using the χ^2^ test.

To determine the sensitivity and specificity of BTP as a predictor of diabetes, a receiver operating characteristic (ROC) curve was used. The chance of positive or negative results in diabetic patients based upon the expression of BTP was evaluated, respectively, by positive and negative likelihood ratios. The significance level was 95%. Statistical analyses were performed using Stata software version 11.0^®^ (USA).

This study was approved by the Research Ethics Committee of the *Faculdade de Medicina do ABC*, under number 295.177, CAAE: 16235413.0.0000.0082.

## RESULTS

A total of 110 patients were recruited for this study: 90 subjects with T2DM and 20 without diabetes serving as the Control Group. The purpose of the control group was to validate the analysis of BTP gene expression.

The Diabetic Group had mean glycated hemoglobin of 8.38% and mean eGFR of 79.1mL/min/1.73m^2^. Of the 90 patients, 48.8% were men and the mean age was 61.3 years. Of the 20 patients in the Control Group, 25% were men and the total mean age was 38.3 years.

Table 1 shows the mean and median expression of BTP observed. Both mean (p=0.08) and median (p=0.70) were higher for the Diabetic Group, compared to the Control Group, but no statistically significant difference between groups was encountered.

**Table 1:** Beta-trace protein expression analysis between groups

Group	Mean (SD)	CV	Median (p.25;p.75)	p value*	p value†
Control	1.07 (5.27)	4.93	2.01 (-2.66; 4.15)	0.158	0.080
T2DM	3.57(5.21)	1.46	2.80 (0.13; 5.42)		

* Student's *t* test;

† Mann-Whitney's test.

SD: standard deviation; CV: coefficient of variation; p.25-p.75: 25 and 75% percentile; T2DM: type 2 *diabetes mellitus.*

**Figure 1:**
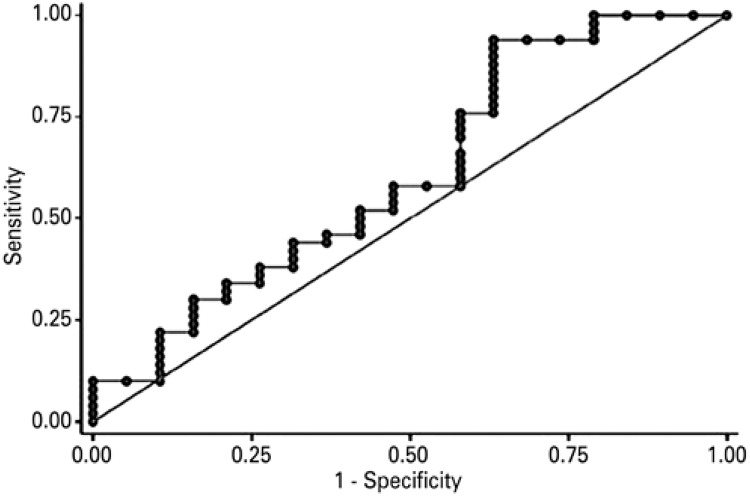
Receiver Operating Characteristic curve of urinary beta-trace protein gene expression between patients with and without type 2 *diabetes mellitus*

Figure 1 shows the diagnostic capacity of BTP as a predictor of kidney damage in people with diabetes, using the ROC curve. Near the cut-off point established by the literature (6.34), BTP as a predictor of kidney damage had a sensitivity of 20%, a specificity of 89.47%, an accuracy of 39.13%, a positive likelihood ratio of 1.90 and a negative likelihood ratio of 0.89, with an area under the curve (AUC) ROC curve of 0.610.

## DISCUSSION

The methodology used in this study is unprecedented in the literature because instead of investigating the concentration of BTP, the variation based upon expression of the gene for BTP was measured. A possible relationship between the presence or absence of change in renal function in diabetics and the expression of BTP was verified.^(^ ^5^ ^,^ ^6^ ^)^

Beta-trace protein is widely found in tissues and body fluids, and increases in the concentration of BTP have been studied as a possible biomarker of reduced glomerular filtration rate. However, as with other markers, the concentration of BTP found showed great variability depending on the method of measurement used, in addition to intra-individual fluctuations. Selvin et al.,^(^ ^7^ ^)^ evaluated the coefficient of variation among the various markers of glomerular filtration rate, such as cystatin C, beta-2 microglobulin and BTP. Of all the markers assessed, BTP, when measured in terms of mass and concentration, showed the greatest intra-individual variability.

This variability is at least partially caused by the existence of various isomers of BTP. The discrepant measurements are the result of changing relative concentrations of each isomer. Furthermore, the differences present in side-chain glycosylation, as well as differences in clearance, are dependent upon the specific fluid (blood, urine or cerebrospinal fluid) where the BTP is encountered.^(^ ^8^ ^,^ ^9^ ^)^ Beta-trace protein in cerebrospinal fluid, with relatively low glycosylation, is excreted by the liver, where the isomers with high glycosylation rates undergo renal excretion.^(^ ^8^ ^)^ The measurement of BTP based on gene expression, rather than on concentration, eliminates variations related to isomers.

Given this methodological advantage, the analysis of the ROC curve showed satisfactory results, taking into account that the value of 0.7 is generally considered as the lower limit for a good marker, and above 0.9 is considered to be excellent. The fact that this analysis, using gene expression, reached a value of 0.61, is promising.

Several studies have verified an increase in the concentration of BTP in diabetic patients who had already presented other signs of reduced renal function.^(^ ^10^ ^,^ ^11^ ^)^ An increase in BTP was also verified in patients with metabolic syndrome and patients who have presented cardiovascular events, suggesting that BTP may also be used as a predictor of diabetes.^(^ ^12^ ^)^ However, none of these studies correlated the presence of diabetes in the absence of kidney disease or other pathological conditions, such as infections or changes in cerebrospinal fluid, with increases in BTP.

## CONCLUSION

Urinary beta-trace protein gene expression in diabetic patients had good sensitivity and specificity. These results are promising, despite their not having achieved a higher area under the curve level.
